# The Role of Genetic Variation in Phenotypic Variability in Loeys–Dietz Syndrome

**DOI:** 10.1155/humu/2861205

**Published:** 2026-09-06

**Authors:** Hussein Abdul Nabi, Luke Dreher, Jack Hartnett, Ashley Crossley, Hussein El Ghandour, Mayowa Osundiji, Hicham El Masry, Chadi Ayoub, Fadi E. Shamoun, Linnea M. Baudhuin

**Affiliations:** ^1^ Department of Cardiovascular Medicine, Mayo Clinic Arizona, Phoenix, Arizona, USA, mayoclinic.org; ^2^ Department of Laboratory Medicine and Pathology, Mayo Clinic, Rochester, Minnesota, USA, mayoclinic.org; ^3^ Department of Clinical Genomics, Mayo Clinic, Rochester, Minnesota, USA, mayoclinic.org

**Keywords:** arrhythmias, genotype–phenotype correlation, Loeys–Dietz syndrome, surgical interventions

## Abstract

**Background:**

Loeys–Dietz syndrome (LDS) is a heritable connective tissue disorder caused by pathogenic variants in genes of the transforming growth factor‐*β* (TGF‐*β*) signaling pathway. Although genotype–phenotype correlations have been suggested, comprehensive comparative data across LDS subtypes remain limited. Improved understanding of these correlations is essential to guide individualized surveillance and management strategies.

**Methods:**

We conducted a retrospective cohort study of adults with genetically confirmed LDS evaluated between 2018 and 2024 across Mayo Clinic sites. Patients with pathogenic, likely pathogenic, or suspicious variants in *TGFBR1*, *TGFBR2*, or *SMAD3* were included. Clinical characteristics, physical examination findings, cardiovascular and noncardiovascular manifestations, surgical interventions, and mortality were compared across genotypes. Categorical variables were analyzed using chi‐square tests and continuous variables using parametric or nonparametric methods as appropriate.

**Results:**

A total of 93 patients were included (29 *TGFBR1*, 33 *TGFBR2*, 31 *SMAD3*). Demographics and mortality did not differ significantly between groups. Patients with *TGFBR1* and *TGFBR2* demonstrated trends toward higher rates of ascending aortic aneurysm and aortic dissection compared with *SMAD3* patients, though these differences were not statistically significant. Renal artery aneurysms were significantly more common in *TGFBR1* patients (13.8%, *p* = 0.010). SMAD3 patients had significantly higher rates of mitral regurgitation (54.8%, *p* = 0.04), peripheral neuropathy (29.0%, *p* = 0.018), and trends toward increased atrial fibrillation and osteoarthritis. A novel association was identified between *TGFBR1* variants and migraine, which was significantly more prevalent in this group (62.1%, *p* = 0.017). The rates of major cardiovascular surgical interventions were high and comparable across all genotypes.

**Conclusion:**

Distinct genotype–phenotype associations exist among LDS subtypes. *TGFBR1* and *TGFBR2* variants are associated with a greater burden of aggressive vascular disease, whereas *SMAD3* variants are linked to mitral valve disease, peripheral neuropathy, and osteoarthritis. We also identified a novel association between *TGFBR1* genotype and migraine. These findings reinforce the importance of comprehensive genetic testing to inform personalized surveillance and management strategies in LDS.

## 1. Introduction

Loeys–Dietz syndrome (LDS) is a connective tissue disorder (CTD) resulting from variants in genes related to the transforming growth factor beta (TGF‐*β*) signaling pathway, including transforming growth factor beta receptors 1 and 2 (*TGFBR1* and *TGFBR2*), small mother against decapentaplegic 2 and 3 *(SMAD2* and *SMAD3*), and transforming growth factor beta 2 and 3 (*TGFB2* and *TGFB3*) [1]. The first described cases of LDS involved variants in the *TGFBR1* and *TGFBR2* genes, and they remain the most frequently involved genes in this syndrome [2, 3]. Initially, LDS was classified into two types depending on craniofacial (Type 1) or skin involvement (Type 2), but there are now six types of LDS based on which gene is involved (*TGFBR1*, *TGFBR2*, *SMAD3*, *TGFB2*, *TGFB3*, and *SMAD2*, respectively). Aberrations in these genes lead to overexpression of the TGF‐*β* pathway and disrupt the structural integrity and function of the extracellular matrix, leading to multisystem abnormalities [1].

There are no consensus clinical criteria for the diagnosis of LDS, and instead diagnosis is suspected in individuals with vascular, skeletal, craniofacial, cutaneous, allergic/inflammatory, ocular, and family history findings. Initially, LDS can be misdiagnosed as Marfan syndrome because of overlapping vascular, cutaneous, and skeletal manifestations. Although patients with LDS typically experience aortic dissections at younger ages and at smaller diameters compared with those with Marfan syndrome [4], there is still phenotypic overlap between these disorders and genetic testing is necessary for accurate diagnosis and risk stratification. Advances in next‐generation sequencing (NGS) technologies have facilitated the identification of novel disease‐causing genes, enabled clinical gene panel testing, and contributed to a deeper understanding of genotype–phenotype correlations in LDS [3].

Establishing these correlations is important for guiding clinical management as early studies suggest different genes and variants can result in variable phenotypic severity. Previous research has shown that *TGFBR2* involvement typically results in milder craniofacial abnormalities compared with *TGFBR1* [5, 6]. Additionally, *SMAD3* variants are uniquely associated with osteoarthritis (and sometimes referred to as aneurysm–osteoarthritis syndrome), and along with *TGFBR1/2* variants, generally correlate with more severe cardiovascular presentations compared with *TGFB2* and *TGFB3* [1, 2]. Patients carrying *TGFBR1* and *TGFBR2* variants accompanied by severe craniofacial features often experience aneurysm ruptures at younger ages and smaller aortic diameters compared with other aneurysm syndromes [5].

In this study, we sought to investigate the association between LDS genotypes and clinical characteristics, physical examination findings, and both cardiac and noncardiac clinical complications. The central aim was to further characterize genotype–phenotype correlations that may better inform patient management.

## 2. Methods

### 2.1. Study Population

This retrospective study analyzed patients diagnosed with LDS between 2018 and 2024 across Mayo Clinic sites in Rochester, Arizona, and Florida. The study cohort was identified using an institutional electronic data retrieval tool, and detailed chart reviews were conducted to confirm the diagnosis.

The study was approved by the Institutional Review Board (IRB) prior to data collection and informed consent was waived because of the retrospective nature of the study. Only patients with genetically confirmed LDS were included. Genetic variants were classified using a five‐tier system: pathogenic (P), likely pathogenic (LP), variant of uncertain significance (VUS), likely benign (LB), or benign (B), according to the 2015 American College of Medical Genetics and Genomics (ACMG) and Association for Molecular Pathology (AMP) joint recommendations [7]. Only variants classified as P, LP, or suspicious VUS (sVUS) were included in the analysis.

Patients younger than 18 years of age were excluded. Because of the small number of identified patients with *TGFB2* and *TGFB3* variants, these individuals were excluded from the analysis. The final study population included only patients with variants in *TGFBR1*, *TGFBR2*, and *SMAD3*.

A comprehensive database was developed from chart reviews and included demographic information, clinical characteristics, comorbidities, and surgical history. Extracted CTD features included scoliosis or kyphosis; pectus deformities (excavatum or carinatum); joint manifestations (hypermobility, dislocations, and tendon rupture); valgus or varus deformities; pes planus; hammer toes; dermatologic features (easy bruising, soft skin); ocular findings (cataracts, exotropia, and ectopia lentis); high‐arched palate; retrognathia; dolichocephaly; malar hypoplasia; dental crowding; hypertelorism; bifid uvula; cleft palate; and down‐slanting palpebral fissures.

Aneurysms and dissections involving the aorta and other vascular territories were included. Recorded surgical interventions included aortic root replacement or repair; mitral, tricuspid, and aortic valve repair or replacement; patent foramen ovale (PFO) closure; and atrial or ventricular septal defect (ASD/VSD) closure.

### 2.2. Study Groups

Patients were divided into three groups: Group 1 included patients with variants in *TGFBR1*; Group 2 included patients with variants in *TGFBR2*, and Group 3 included patients with variants in *SMAD3*.

### 2.3. Study Outcomes

The primary objective of this study was to investigate genotype–phenotype correlations by evaluating whether specific genetic variants were associated with differences in physical examination findings, aortic and nonaortic arterial complications including aneurysms and dissections, surgical interventions, and mortality.

### 2.4. Statistical Analysis

Statistical analyses were performed using the Statistical Package for the Social Sciences (SPSS). Categorical variables are reported as frequencies and percentages (*n*, %) and were analyzed using chi‐square tests. Continuous variables were assessed for normality using the Shapiro–Wilk test and analyzed using independent *t*‐tests or Mann–Whitney *U* tests, as appropriate. Continuous variables are reported as mean ± standard deviation (SD) or median [interquartile range (IQR)], depending on data distribution. Statistical significance was defined as *p* < 0.05.

## 3. Results

### 3.1. Genetic Testing

**Figure 1:**
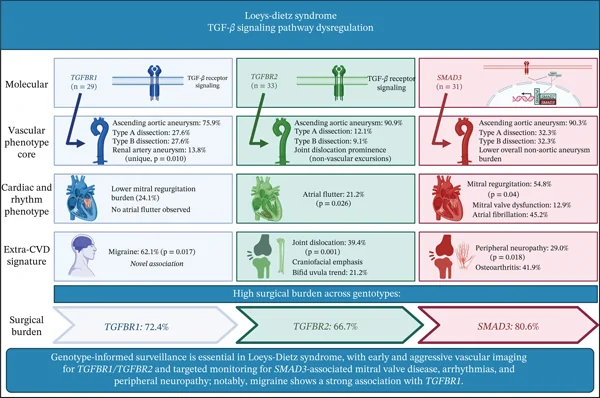
Genotype–phenotype correlates across TGF‐*β* pathway genes in Loeys–Dietz syndrome (TGFBR1, TGFBR2, SMAD3). TGF‐*β* signaling pathway dysregulation and genotype‐specific clinical signatures in Loeys–Dietz syndrome. In this retrospective cohort of 93 adults with genetically confirmed LDS evaluated from 2018–2024 (TGFBR1 *n* = 29; TGFBR2 *n* = 33; SMAD3 *n* = 31), vascular, cardiac/rhythm, and extracardiovascular phenotypes differed across genotypes. TGFBR1 and TGFBR2 demonstrated a high burden of ascending aortic aneurysm (75.9% and 90.9%, respectively), with renal artery aneurysm enrichment in TGFBR1 (13.8%, *p* = 0.010). SMAD3 showed higher MR prevalence (54.8%, *p* = 0.04) and a greater burden of peripheral neuropathy (29.0%, *p* = 0.018), with osteoarthritis remaining prominent. Arrhythmic patterns varied, including increased atrial flutter in TGFBR2 (21.2%, *p* = 0.026) and a trend toward higher atrial fibrillation in SMAD3 (45.2%). A novel association was observed between TGFBR1 variants and migraine (62.1%, *p* = 0.017). Major cardiovascular surgical intervention rates were high across genotypes, including aortic root replacement (TGFBR1 72.4%; TGFBR2 66.7%; SMAD3 80.6%). Collectively, these data support genotype‐informed surveillance, earlier aggressive vascular imaging for TGFBR1/TGFBR2 and targeted monitoring for SMAD3‐associated mitral valve disease, arrhythmias, and peripheral neuropathy. Created in BioRender. Dreher, L. (2026) https://BioRender.com/hv1jfcu.

A total of 93 individuals diagnosed with LDS were identified through retrospective chart review. All diagnoses were confirmed by genetic testing. Variants were identified in *TGFBR1*, *TGFBR2*, and *SMAD3*. Thirty‐one patients carried *SMAD3* variants, classified as sVUS (*n* = 1), LP (*n* = 18), and P variants (*n* = 12). There were 29 patients who had *TGFBR1* variants, including 3 sVUS, 20 LP variants, and 6 P variants. Thirty‐three patients carried *TGFBR2* variants, consisting of 20 LP variants and 13 P variants. Patients were categorized into three genetic groups: Group 1 (*TGFBR1* variants), Group 2 (*TGFBR2* variants), and Group 3 (*SMAD3* variants). Figure 1 serves as the central illustration, summarizing the key findings of the study.

### 3.2. Demographics

Demographic characteristics were similar across the three groups. Gender distribution did not differ significantly among groups (*p* = 0.193), with comparable representation of males and females. Racial distribution was also similar, with 82 patients (88.2%) identifying as White (*p* = 0.351).

Mortality rates did not significantly differ between groups (*p* = 0.428). Death occurred in 10.3% of patients with *TGFBR1* variants, 6.1% of those with *TGFBR2* variants, and 16.1% of patients with *SMAD3* variants. Demographic characteristics and comorbidities are summarized in Table 1.

**Table 1:** Demographic characteristics and comorbidities.

Feature	Group 1 (*n* = 29)	Group 2 (*n* = 33)	Group 3 (*n* = 31)	*p*
Gender				0.193
Female	18 (62.1%)	16 (48.5%)	12 (38.7%)	
Male	11 (37.9%)	17 (51.5%)	19 (61.3%)	
Race				0.351
White	27 (93.1%)	27 (81.8%)	28 (90.3%)	
Non‐White	2 (6.9%)	6 (18.2%)	3 (9.7%)	
Death	3 (10.3%)	2 (6.1%)	5 (16.1%)	0.428
DM	0 (0%)	2 (6.1%)	4 (12.9%)	0.126
HTN	11 (63.6%)	21 (63.6%)	18 (58.1%)	0.108
Migraine	18 (62.1%)	5 (15.2%)	7 (22.6%)	0.017
HLD	5 (17.2%)	12 (36.4%)	14 (45.2%)	0.065
Stroke	3 (10.3%)	4 (12.1%)	5 (16.1%)	0.789
MI	0 (0%)	2 (6.1%)	3 (9.7%)	0.246
HF	4 (13.8%)	6 (18.2%)	10 (32.3%)	0.186
Osteoarthritis	7 (24.1%)	11 (33.3%)	13 (41.9%)	0.344
Peripheral neuropathy	1 (3.4%)	4 (12.1%)	9 (29.0%)	0.018

Abbreviations: DM, diabetes mellitus; HF, heart failure; HLD, hyperlipidemia; HTN, hypertension; MI, myocardial infarction.

### 3.3. Comorbidities

Multiple comorbid conditions were evaluated across the three groups. The only comorbidity to reach a statistically significant association was migraine. Migraines were significantly more prevalent among patients with *TGFBR1* variants (62.1%) compared with those carrying *TGFBR2* (15.2%) or *SMAD3* variants (22.6%) (*p* = 0.017). When stratified by sex, migraine remained more frequent among females in all three genetic groups: among *TGFBR1* patients, migraine occurred in 14 of 18 females (77.8%) and 4 of 11 males (36.4%); among *TGFBR2* patients, in 4 of 16 females (25.0%) and 1 of 17 males (5.9%); and among *SMAD3* patients, in 5 of 12 females (41.7%) and 2 of 19 males (10.5%).

In relation to risk factors for vascular disease, hypertension was most common in patients with *TGFBR2* variants (63.6%), compared with 37.9% in *TGFBR1* and 58.1% in *SMAD3* patients; however, this difference did not reach statistical significance (*p* = 0.108). Diabetes mellitus was observed most frequently in the *SMAD3* group (12.9%), compared with 0% in *TGFBR1* and 6.1% in *TGFBR2* patients (*p* = 0.126). Hyperlipidemia occurred most frequently in the *SMAD3* group (45.2%), compared with 36.4% in *TGFBR2* and 17.2% in *TGFBR1* patients; this trend approached but did not reach statistical significance (*p* = 0.065). Body mass index (BMI) did not differ significantly among the three genetic groups (*p* = 0.959).

The prevalence of stroke was highest among *SMAD3* patients (16.7%), followed by *TGFBR2* (12.1%) and *TGFBR1* (10.3%) groups (*p* = 0.789). Heart failure (HF) was observed in 13.8% of *TGFBR1*, 18.2% of *TGFBR2*, and 32.3% of *SMAD3* patients, showing a trend toward higher prevalence in *SMAD3* (*p* = 0.186).

Osteoarthritis was most prevalent among *SMAD3* patients (41.9%), followed by *TGFBR2* (33.3%) and *TGFBR1* (24.1%) groups; however, no statistically significant difference was observed (*p* = 0.344). Peripheral neuropathy differed significantly across genetic groups (*p* = 0.018), occurring most frequently in the *SMAD3* group (29.0%), compared with 3.4% in *TGFBR1* and 12.1% in *TGFBR2* patients. Of the 14 patients with peripheral neuropathy, only 2 had concomitant diabetes mellitus, both of whom had *SMAD3* variants; these represented 2 of the 9 *SMAD3* patients with peripheral neuropathy.

### 3.4. Physical Exam Findings

Physical examination findings were compared across the three genetic groups. The prevalence of scoliosis was similar among groups, with no statistically significant difference observed (p = 0.834). Pectus deformities were more common in patients with *TGFBR1* variants (51.7%), compared with 39.4% in the *TGFBR2* group and 25.8% in the *SMAD3* group. However, this trend did not reach significance (*p* = 0.119).

The presence of pes planus was comparable across all groups (*p* = 0.460). Joint hypermobility was most frequently observed in patients with *SMAD3* variants (45.2%), compared with 20.7% in *TGFBR1* and 24.2% in *TGFBR2* patients, approaching but not reaching statistical significance (*p* = 0.078). In contrast, joint dislocations were significantly more prevalent in the *TGFBR2* group, affecting 29.4% of patients, compared with 6.9% in *TGFBR1* and 9.7% in *SMAD3* patients (*p* = 0.001).

Cutaneous findings were comparable across all genetic groups (p = 0.963). Ophthalmologic manifestations, including cataracts and ectopia lentis, were more commonly observed in patients with *SMAD3* variants (29.0%) compared with *TGFBR1* (6.9%) and *TGFBR2* (24.2%) patients, though this difference did not reach statistical significance (*p* = 0.083).

Bifid uvula was most frequently identified in patients with *TGFBR2* variants (21.2%), compared with 10.3% in the *TGFBR1* group and 3.2% in the *SMAD3* group (*p* = 0.080). Downslanting palpebral fissures were also more common in the *TGFBR2* group (15.2%) compared with *TGFBR1* (3.4%) and *SMAD3* (3.2%) groups, but this trend was not statistically significant (*p* = 0.118). Detailed physical examination findings are summarized in Table 2.

**Table 2:** Physical examination findings.

Feature	Group 1 (*n* = 29)	Group 2 (*n* = 33)	Group 3 (*n* = 31)	*p*
Scoliosis	13 (44.8%)	17 (51.5%)	16 (51.6%)	0.834
Pectus	15 (51.7%)	13 (39.4%)	8 (25.8%)	0.119
Flat feet	10 (34.5%)	7 (21.2%)	10 (32.3%)	0.46
Valgus/varum	5 (17.2%)	7 (21.9%)	3 (9.7%)	0.418
Joint condition	8 (27.6%)	17 (51.5%)	14 (45.2%)	0.147
Joint hypermobility	6 (20.7%)	8 (24.2%)	14 (45.2%)	0.078
Joint dislocation	2 (6.9%)	13 (39.4%)	3 (9.7%)	0.001
Skin condition	7 (24.1%)	7 (21.2%)	7 (22.6%)	0.963
Eyes	2 (6.9%)	8 (24.2%)	9 (29.0%)	0.083
High arched	4 (13.8%)	11 (33.3%)	6 (19.4%)	0.161
Retrognathia	2 (6.9%)	6 (18.2%)	1 (3.2%)	0.107
Dolichocephaly	2 (6.9%)	4 (12.1%)	3 (9.7%)	0.786
Malar hypoplasia	2 (6.9%)	8 (24.2%)	5 (16.1%)	0.18
Dental crowding	5 (17.2%)	5 (15.2%)	2 (6.5%)	0.41
Hypertelorism	4 (13.8%)	4 (12.1%)	3 (9.7%)	0.884
Bifid uvula	3 (10.3%)	7 (21.2%)	1 (3.2%)	0.08
Cleft palate	0 (0%)	0 (0%)	1 (3.2%)	0.364
Downslanting palpebral fissures	1 (3.4%)	5 (15.2%)	1 (3.2%)	0.118

### 3.5. Cardiovascular Findings

Cardiovascular manifestations were assessed across the three genetic groups. Ascending aortic aneurysms were observed in 75.9% of *TGFBR1*, 90.9% of *TGFBR2*, and 90.3% of *SMAD3* patients (*p* = 0.164), suggesting a trend toward lower prevalence in the *TGFBR1* group. Abdominal aortic aneurysms (AAA) were relatively similar across groups (27.6% *TGFBR1*, 27.3% *TGFBR2*, 29.0% *SMAD3*; *p* = 0.986).

The occurrence of aortic dissections showed some genotype‐specific patterns. Type A dissections were observed in 27.6% of *TGFBR1*, 12.1% of *TGFBR2*, and 22.6% of *SMAD3* patients (*p* = 0.301), whereas type B dissections were seen in 27.6% of *TGFBR1*, 9.1% of *TGFBR2*, and 16.1% of *SMAD3* patients (*p* = 0.154). Although these differences were not statistically significant, they suggest a trend toward higher dissection rates in the *TGFBR1* group for both Type A and B dissections. Overall dissection presence occurred in 27.6% of *TGFBR1*, 18.2% of *TGFBR2*, and 32.3% of *SMAD3* patients (*p* = 0.422), indicating a slightly higher burden in *SMAD3* and *TGFBR1* patients.

Other arterial dissections, including vertebral, carotid, coronary, innominate, iliac, subclavian, cerebral, splenic, and mesenteric arteries, were uncommon and did not differ significantly across groups (all *p* > 0.05). Regarding aneurysm distribution, most vascular territories were similarly affected across genotypes, except for renal artery aneurysms, which were significantly more frequent in *TGFBR1* patients (13.8%) compared with 0% in *TGFBR2* and *SMAD3* groups (*p* = 0.010). Detailed aneurysm and dissection distributions are presented in Table 3.

**Table 3:** Aneurysm and dissection distributions.

Feature	Group 1 (*n* = 29)	Group 2 (*n* = 33)	Group 3 (*n* = 31)	*p*
Ascending aorta aneurysm	22 (75.9%)	30 (90.9%)	28 (90.3%)	0.164
Abdominal aortic aneurysm	8 (27.6%)	9 (27.3%)	9 (29.0%)	0.986
Type A	8 (27.6%)	4 (12.1%)	7 (22.6%)	0.301
Type B	8 (27.6%)	3 (9.1%)	5 (16.1%)	0.154
Dissection presence	8 (27.6%)	6 (18.2%)	10 (32.3%)	0.422
Vertebral artery dissection	1 (3.4%)	3 (9.1%)	4 (12.9%)	0.423
Carotid artery dissection	5 (17.2%)	1 (3.0%)	4 (12.9%)	0.176
Renal artery dissection	1 (3.4%)	0 (0%)	0 (0%)	0.328
Iliac artery dissection	1 (3.4%)	1 (3.0%)	1 (3.2%)	0.996
Coronary artery dissection	2 (6.9%)	0 (0%)	2 (6.5%)	0.316
Innominate artery dissection	2 (6.9%)	1 (3.0%)	1 (3.2%)	0.708
Aneurysm presence	17 (58.6%)	16 (48.5%)	19 (61.3%)	0.552
Carotid artery aneurysm	5 (17.2%)	4 (12.1%)	4 (12.9%)	0.826
Vertebral artery aneurysm	1 (3.4%)	1 (3.0%)	1 (3.2%)	0.996
Splenic artery aneurysm	1 (3.4%)	2 (6.1%)	3 (9.7%)	0.614
Mesenteric artery aneurysm	6 (20.7%)	3 (9.1%)	3 (9.7%)	0.32
Renal artery aneurysm	4 (13.8%)	0 (0%)	0 (0%)	0.01
Iliac artery aneurysm	5 (17.2%)	4 (12.1%)	10 (32.3%)	0.119
Subclavian artery aneurysm	2 (6.9%)	3 (9.1%)	2 (6.5%)	0.912
Cerebral artery aneurysm	7 (24.1%)	8 (24.2%)	9 (29.0%)	0.881
Coronary artery aneurysm	5 (17.2%)	1 (3.0%)	3 (9.7%)	0.168

### 3.6. Valvular Abnormalities and Cardiovascular Surgery

Regarding valvular abnormalities, mitral regurgitation (MR) was present in 24.1% of *TGFBR1*, 33.3% of *TGFBR2*, and 54.8% of *SMAD3* patients, showing a statistically significant difference between groups (*p* = 0.04), with a trend toward more MR in *SMAD3* patients. Mitral valve prolapse (MVP) was observed in 24.1% of *TGFBR1*, 45.5% of *TGFBR2*, and 32.3% of *SMAD3* patients (*p* = 0.202). Tricuspid regurgitation (TR) occurred in 10.3% of *TGFBR1*, 12.1% of *TGFBR2*, and 3.2% of *SMAD3* patients (*p* = 0.412), whereas bicuspid aortic valve was rare, observed in 10.3%, 9.1%, and 6.5% of patients across groups, respectively (*p* = 0.859). Aortic regurgitation was present in roughly half of patients in each group (51.7% *TGFBR1*, 45.5% *TGFBR2*, 51.6% *SMAD3*; *p* = 0.848).

Mitral valve replacement (MVR) was performed in 6.9% of *TGFBR1*, 9.1% of *TGFBR2*, and 12.9% of *SMAD3* patients (*p* = 0.727), whereas tricuspid valve replacement (TVR) was less frequent, occurring in 17.2% of *TGFBR1*, 6.1% of *TGFBR2*, and 3.2% of *SMAD3* patients, showing a trend toward more TVR in *TGFBR1* (*p* = 0.125). Aortic valve replacement (AVR) was performed in 44.8% of *TGFBR1*, 39.4% of *TGFBR2*, and 54.8% of *SMAD3* patients (*p* = 0.457), and aortic root replacement (ARR) was the most common intervention overall, observed in 72.4% of *TGFBR1*, 66.7% of *TGFBR2*, and 80.6% of *SMAD3* patients (*p* = 0.449). Additional procedures, including ASD, VSD, and PFO closure, were performed in 27.6% of *TGFBR1*, 15.2% of *TGFBR2*, and 16.1% of *SMAD3* patients (*p* = 0.399).

Overall, although trends in valvular abnormalities were identified, particularly higher rates of MR in *SMAD3*, differences in rates of most surgical interventions and valvular abnormalities did not reach statistical significance. Detailed cardiovascular surgery and valvular abnormality distributions are presented in Table 4.

**Table 4:** Cardiovascular surgery and valvular abnormality distributions.

Variable	Group 1 (*n* = 29)	Group 2 (*n* = 33)	Group 3 (*n* = 31)	*p*
MR	7 (24.1%)	11 (33.3%)	17 (54.8%)	0.04
TR	3 (10.3%)	4 (12.1%)	1 (3.2%)	0.412
Bicuspid aortic valve	3 (10.3%)	3 (9.1%)	2 (6.5%)	0.859
MVP	7 (24.1%)	15 (45.5%)	10 (32.3%)	0.202
Aortic regurgitation	15 (51.7%)	15 (45.5%)	16 (51.6%)	0.848
MVR	2 (6.9%)	3 (9.1%)	4 (12.9%)	0.727
TVR	5 (17.2%)	2 (6.1%)	1 (3.2%)	0.125
AVR	13 (44.8%)	13 (39.4%)	17 (54.8%)	0.457
ARR	21 (72.4%)	22 (66.7%)	25 (80.6%)	0.449
ASD/VSD/PFO Closure	8 (27.6%)	5 (15.2%)	5 (16.1%)	0.399

Abbreviations: ARR, aortic root replacement; ASD, atrial septal defect; AVR, aortic valve replacement; MR, mitral regurgitation; MVP, mitral valve prolapse; MVR, mitral valve replacement; PFO, patent foramen ovale; TR, tricuspid regurgitation; TVR, tricuspid valve replacement; VSD, ventricular septal defect.

### 3.7. Cardiac Arrhythmias

The prevalence of arrhythmias varied across the three groups. AF was present in 27.6% of *TGFBR1*, 24.2% of *TGFBR2*, and 45.2% of *SMAD3* patients, showing a trend toward higher AF prevalence in *SMAD3* (*p* = 0.164). Atrial flutter was more frequent in *TGFBR2* (21.2%) compared with *TGFBR1* (0%) and *SMAD3* (9.7%), reaching statistical significance (*p* = 0.026), whereas the combined “category one” arrhythmia group occurred in 27.6%, 30.3%, and 45.2% of patients, respectively (*p* = 0.296).

Supraventricular tachycardia (SVT) was observed in 24.1% of *TGFBR1*, 9.1% of *TGFBR2*, and 19.4% of *SMAD3* patients (*p* = 0.272), whereas ventricular tachycardia (VT) occurred in 10.3%, 18.2%, and 22.6% across groups (*p* = 0.447). Ventricular fibrillation or VT (VF/VF tach) was most common in *SMAD3* (29.0%), compared with 10.3% in *TGFBR1* and 21.2% in *TGFBR2*, showing a trend toward increased risk in *SMAD3* (*p* = 0.198). Overall, atrial flutter and VF/VF showed notable differences among groups, whereas other arrhythmias demonstrated trends without reaching statistical significance. Detailed arrhythmia distributions are presented in Table 5.

**Table 5:** Arrhythmia distributions.

Arrhythmia	Group 1 (*n* = 29)	Group 2 (*n* = 33)	Group 3 (*n* = 31)	*p*
Atrial fibrillation	8 (27.6%)	8 (24.2%)	14 (45.2%)	0.164
Atrial flutter	0 (0.0%)	7 (21.2%)	3 (9.7%)	0.026
Category one^a^	8 (27.6%)	10 (30.3%)	14 (45.2%)	0.296
SVT	7 (24.1%)	3 (9.1%)	6 (19.4%)	0.272
VT	3 (10.3%)	6 (18.2%)	7 (22.6%)	0.447
VF/VT	3 (10.3%)	7 (21.2%)	9 (29.0%)	0.198

Abbreviations: SVT, supraventricular tachycardia; VF, ventricular fibrillation; VT, ventricular tachycardia.

^a^Category one refers to patients having atrial arrhythmia (atrial fibrillation or atrial flutter).

## 4. Discussion

In this study genotype–phenotype correlations were identified among 93 LDS patients, particularly highlighting the significance of gene‐specific differences (*TGFBR1*, *TGFBR2*, *SMAD3*). Overall, in keeping with prior publications, our data suggest that *TGFBR1* and *TGFBR2* patients experience more aortic and nonaortic vascular complications in comparison to *SMAD3* patients. Although vascular disease was less common, *SMAD3* patients tended to experience more osteoarthritis and MR.

There was no difference in mortality between *TGFBR1*, *TGFBR2*, and *SMAD3* LDS patients. Among LDS types, *TGFBR1*, *TGFBR2*, and *SMAD3* mutations drive more aggressive phenotypes with earlier vascular disease compared with milder *TGFB1* and *TGFB2* mutations [1]. Although *TGFB1* and *TGFB2* groups were not studied because of low numbers, lower mortality among these patients would be expected.

Although not statistically significant, ascending aortic aneurysms were more frequently seen in *TGFBR2* and *SMAD3* patients compared with the *TGFBR1* cohort (75.9% vs. 90.9% vs. 90.3%, *p* = 0.164). Similarly, both Type A and Type B aortic dissections were numerically most common in the *TGFBR1* group followed by *SMAD3* then *TGFBR2* (Type A: 27.6% vs. 12.1% vs. 22.6%, Type B: 27.6% vs. 9.1% vs. 16.1%). Previous literature has documented the propensity of *TGFBR1* and *TGFBR2* variants for early‐onset severe vascular events, even at smaller aortic dimensions [5]. In larger registries, over 40% of patients with *TGFBR1/2* variants experienced aortic dissections, with some dissections occurring at diameters below 45 mm, especially in female *TGFBR2* carriers [3].

Our findings correspond well with the current understanding of LDS pathophysiology, whereby loss of function mutations in the TGF‐*β* receptor genes lead to paradoxical activation of TGF‐*β* signaling. Numerous different hypotheses regarding the underlying mechanism for paradoxical activity have been proposed including loss of negative feedback leading to enhanced pathway activity and paracrine TGF‐*β* signaling [1]. Regardless, repeated studies have demonstrated a high propensity for early‐onset aortic aneurysms and dissections in *TGFBR1* and *TGFBR2* patients [3, 6].

In comparison to prior data, our cohort showed numerically lower rates of aortic aneurysms and dissections among *SMAD3* patients. An international *SMAD3* cohort (Montalcino Aortic Consortium) reported aortic events in 37% of their cases, in which 70% of those were dissections [7]. The median age of onset of aortic event in this cohort was 47 years, notably higher than *TGFBR1* and *TGFBR2* patients in other studies.

Vascular and valvular surgery was required at similar rates across the three genetic subtypes. Although aortic valve replacement was necessary in approximately half of the patients in each cohort, aortic root replacement was required in almost 72.4% of *TGFBR1* patients, 66.7% of *TGFBR2* patients, and 80.6% of *SMAD3* patients. The high rate of aortic surgery has been well established among *TGFBR1* and *TGFBR2* patients. In a seminal study of 90 *TGFBR1* or *TGFBR2* patients published in the *New England Journal of Medicine*, 59 patients required vascular surgery involving the aorta or its major branches and the median age of first surgery was 16.9 years [5].

Although rates of aortic aneurysm and dissection were lower, aortic root replacement rates in our *SMAD3* group are higher than previously reported. This is in keeping with longitudinal data which indicate *SMAD3* patients often require prophylactic aortic surgery—in one series, 50% of patients underwent elective root replacement within approximately 10 years of diagnosis [8]. Our findings are broadly consistent with previous studies linking *SMAD3* variants to vascular phenotypes, likely due to impaired vascular integrity stemming from abnormal vascular smooth muscle cell function and disrupted extracellular matrix remodeling [9–11]. Aortas with *SMAD3* variants show paradoxical TGF‐B activation, where impaired *SMAD* signaling results in ligand overexpression and excessive noncanonical pathway activity, factors that likely contribute to the arterial wall vulnerability and aneurysm formation [1, 7].

Our data also supports the association between *SMAD3* mutations and mitral valve disease. Although early‐onset ascending aortic disease predominates in *TGFBR1* and *TGFBR2* LDS, mitral valve disease is often the most common cardiovascular manifestation among *SMAD3* patients. In one study, 84% of *SMAD3* patients had a cardiovascular abnormality −58% patients hadMVP followed by 42% with aortic disease [12]. Mitral valve disease rates have varied in prior publications from 19% to 58% [7, 12]. Among *SMAD3* patients in our study, 54.8% had MR compared with 24.1% and 33.3% among *TGFBR1* and *TGFBR2* patients, respectively (*p* = 0.04). Although not statistically significant, higher rates of MVR were also observed in the *SMAD3* group (12.9% in *SMAD3* vs. 6.9% in *TGFBR1* and 9.1% in *TGFBR2*).

In keeping with the propensity for MR, atrial fibrillation was most common among *SMAD3* patients (45.2% in *SMAD3* vs. 27.6% in *TGFBR1* and 24.2% in *TGFBR2).* A previous study in patients with LDS demonstrated an association between MR and increased LAVI, which may contribute to the increased risk of AF observed in this population [13]. Thus, the higher burden of AF among SMAD3 patients may, at least in part, reflect their greater prevalence of mitral valve disease rather than a direct genotype‐specific association. *TGFBR1*, *TGFBR2* and *SMAD3* mutations all affect downstream activity of the canonical and noncanonical TGF‐*β* pathway. From a surface level it is unclear why *SMAD3* mutations lead to more mitral valve disease. Further research into the differential mechanistic effects of *SMAD3* mutations on the TGF‐*β* pathway is required to untangle its predisposition for mitral valve involvement.

Previously named aneurysms‐osteoarthritis syndrome, the association between *SMAD3* mutations and early‐onset osteoarthritis has been well established [9]. Prior publications have reported osteoarthritis rates of up to 92% [12]. Notably, osteoarthritis tends to develop at a young age and often involves multiple unusual joints [9, 12]. Although not statistically significant, osteoarthritis was more common in our *SMAD3* group compared with *TGFBR1* and *TGFBR2* patients (41.9% vs. 24.1% vs. 33.3%, *p* = 0.344). However, the osteoarthritis rate in our *SMAD3* group (41.9%) is lower than reported in the existing literature.

There was a significant difference in peripheral neuropathy rates across the groups (*p* = 0.018). Peripheral neuropathy was found most frequently in the *SMAD3* group (29.0%) compared with *TGFBR1* patients (3.4%) and *TGFBR2* patients (12.1%). Early studies of families with *SMAD3* mutations, even prior to it being classified as a form of LDS, reported peripheral neuropathy as a frequent manifestation [14]. The form of neuropathy described is an axonal and sensory neuropathy similar to that of Type II Charcot–Marie–Tooth. Although the links between *SMAD3* and peripheral neuropathy are well characterized, no association between *TGFBR1*, *TGFBR2* mutations, and peripheral neuropathy has been established to date. Our data are in keeping with this.

The higher prevalence of migraine among *TGFBR1* patients represents a novel genotype–phenotype association. Migraine was significantly more prevalent (*p* = 0.017) among *TGFBR1* patients (62.1%) compared with *TGFBR2* patients (15.2%) or *SMAD3* patients (22.6%). When stratified by sex, migraine remained more frequent among females in all three genetic groups. Thus, the higher prevalence of migraine in the *TGFBR1* group was not solely attributable to the female predominance in that group, although females accounted for the majority of affected patients.

No prior studies have identified a link between LDS genotype and migraine. *TGFBR1* mutations are known to cause nonaortic arterial disease, including aneurysms and tortuosity [15, 16]. Intracranial involvement could theoretically precipitate chronic headache in this cohort compared with other genotypes. Although a recent study did determine that 75% of all nonaortic arterial disease in LDS patients involves the arch vessels or cerebral circulation, the authors did not identify any significant variation in the rate of nonaortic arterial disease between genetic subtypes [17]. Another plausible mechanism for the propensity for headache among *TGFBR1* patients is increased rates of cervical spinal disease. Although our data did not identify any significant differences in rates of skeletal deformities between genotypes, prior publications report increased rates of cervical spinal instability in *TGFBR1* patients [16, 18].

Overall, our data are in keeping with prior literature that there are strong genotype–phenotype associations among different LDS subtypes. This emphasizes the value of comprehensive genetic testing in patients with LDS to aid individualized surveillance and management based on genotype.

### 4.1. Limitations

This study has several limitations. Firstly, it involved patients from multiple Mayo Clinic sites within a single tertiary referral healthcare system, potentially restricting the generalizability of the results to broader patient populations with differing management practices and protocols. Because this cohort was derived from a multicenter tertiary referral system rather than a population‐based or genetic registry, it may preferentially represent patients with more complex or severe disease, and differences in referral patterns and genetic ascertainment may therefore result in a distribution of genetic variants that differs from that reported in the general LDS population. Additionally, despite thorough validation efforts using ICD‐10 coding and manual database reviews, there remains the possibility of inaccuracies or misclassification of clinical conditions and comorbidities. Finally, the relatively modest sample size in the setting of this rare, albeit clinically important condition limits statistical power, potentially affecting the ability to detect significant differences among the genetic subtypes.

## 5. Conclusion

The phenotypic variability across genetic subtypes underscores the necessity of individualized surveillance strategies in patients with LDS. *TGFBR1* and *TGFBR2* patients may benefit from earlier and more intensive imaging monitoring for early‐onset aggressive vascular disease, whereas *SMAD3* patients require close attention to mitral valvular disease, peripheral neuropathy, and osteoarthritis. In addition, we report a novel association between *TGFBR1* genotype and migraine. Future studies investigating specific genetic variants in larger cohorts are needed to improve our understanding of underlying mechanisms and refine personalized clinical management strategies for patients with LDS.

## Author Contributions

Hussein Abdul Nabi and Luke Dreher contributed equally to this work and share first authorship.

## Funding

No funding was received for this manuscript.

## Ethics Statement

This study was approved by the Mayo Clinic Institutional Review Board (IRB).

## Conflicts of Interest

The authors declare no conflicts of interest.

## Data Availability

The data that support the findings of this study are available from the corresponding author upon reasonable request.
